# Personal

**Published:** 1904-11

**Authors:** 


### PERSONAL.

Dr. John R. Pratt, of Manchester, and Dr. W. Scott Hicks, of Bristol, celebrated the termination of fifty years of active practice of their profession at the quarterly meeting of the Ontario County Medical Society, held at Canandaigua, N. Y., October 11, 1904. Remarks appropriate to the occasion were made and the exercises closed with a banquet at the Hotel Canandaigua. Dr. Roswell Park, of Buffalo, attended the meeting and presented a paper.

Dr. A. H. Briggs, of Buffalo, was elected first vice-president of The Association of Military Surgeons of the United States at its recent annual meeting held at Saint Louis.

Dr. Brooks H. Wells, New York, has been appointed attending gynecologist to Saint Vincent's Hospital in that city.

Dr. John B. Roberts, Philadelphia, announces the removal of his offices and residence to 313 South Seventeenth street. Hours: 8 a. m. to 12 m.

Dr. E. C. Dudley, of Chicago, president of the American Gynecological Society, has lately returned from a trip to the Pacific Coast. He was the guest of the Pierce County Medical Society at its meeting held at Tacoma, Wash., in September, and delivered a lecture on Surgical operations and pathologic conditions, which was beautifully illustrated by lantern slides.

Frank A. Ruf, Esq., of Saint Louis, president of the Antikamnia Chemical Company, who has recently been touring in Europe, has returned to his home in fine health and spirits. He extended his tour to include the Land of the Midnight Sun of which he speaks in enthusiastic terms.

